# PET Tracers for Imaging Cardiac Function in Cardio-oncology

**DOI:** 10.1007/s11886-022-01641-4

**Published:** 2022-01-13

**Authors:** James M. Kelly, John W. Babich

**Affiliations:** 1 Division of Radiopharmaceutical Sciences and Molecular Imaging Innovations Institute (MI3), Weill Cornell Medicine, Belfer Research Building, Room BB-1604, 413 East 69th St, New York, NY 10021, USA; 2 Citigroup Biomedical Imaging Center, Weill Cornell Medicine, New York, NY 10021, USA; 3 Sandra and Edward Meyer Cancer Center, Weill Cornell Medicine, New York, NY 10021, USA

**Keywords:** Cardiotoxicity, Positron emission tomography, Fibroblast activation protein, Metabolic imaging, Sympathetic innervation

## Abstract

**Purpose of Review:**

Successful treatment of cancer can be hampered by the attendant risk of cardiotoxicity, manifesting as cardiomyopathy, left ventricle systolic dysfunction and, in some cases, heart failure. This risk can be mitigated if the injury to the heart is detected before the onset to irreversible cardiac impairment. The gold standard for cardiac imaging in cardio-oncology is echocardiography. Despite improvements in the application of this modality, it is not typically sensitive to subclinical or early-stage dysfunction. We identify in this review some emerging tracers for detecting incipient cardiotoxicity by positron emission tomography (PET).

**Recent Findings:**

Vectors labeled with positron-emitting radionuclides (e.g., carbon-11, fluorine-18, gallium-68) are now available to study cardiac function, metabolism, and tissue repair in preclinical models. Many of these probes are highly sensitive to early damage, thereby potentially addressing the limitations of current imaging approaches, and show promise in preliminary clinical evaluations.

**Summary:**

The overlapping pathophysiology between cardiotoxicity and heart failure significantly expands the number of imaging tools available to cardio-oncology. This is highlighted by the emergence of radiolabeled probes targeting fibroblast activation protein (FAP) for sensitive detection of dysregulated healing process that underpins adverse cardiac remodeling. The growth of PET scanner technology also creates an opportunity for a renaissance in metabolic imaging in cardio-oncology research.

## Introduction

With over 20 million cancer survivors predicted to be living in the USA by 2026 [1], it is increasingly important to consider the longer-term impact of cancer therapy. Many survivors face a higher risk of death from cardiovascular causes than from cancer recurrence [2]. This is particularly true for survivors of childhood cancer, who exhibit a significantly higher incidence of premature cardiovascular disease than appropriately matched population samples [3–5]. Anthracycline chemotherapy is effective against a broad range of cancers, and its use to treat leukemia, lymphoma, and other solid tumors has contributed to dramatic increases in survival rates for pediatric patients [6]. More than 50% of children with cancer now receive an anthracycline as part of their treatment [3]. However, anthracyclines, such as doxorubicin, are associated with cardiotoxicity [6–8], which can manifest as cardiomyocyte hypertrophy, with progression to cardiomyopathy and eventually heart failure [3]. Subclinical left ventricular abnormalities, such as decreased mass and wall thickness or contractility, are evident in as many as 60% of survivors of pediatric cancer within 6 years of the end of treatment [3, 9]. Doxorubicin cardiotoxicity is dose-dependent [10] and exacerbated by adjuvant therapy and chest irradiation [11, 12]. Acute cardiotoxicity is rare (< 1%) and generally reversible, while early-onset chronic progressive toxicity, which develops during treatment, and late-onset chronic progressive toxicity arising after therapy are often irreversible [2]. Adverse cardiac effects are classified as structural or functional, and these two may be severe and decoupled, especially in early stages of damage [13]. Children are particularly susceptible to the detrimental effects of anthracyclines: approximately 7% of survivors of childhood cancer treated with anthracyclines develop heart failure within 30 years of cancer diagnosis [3], and the risk doubles in those survivors treated with both anthracyclines and external beam radiotherapy [12].

Breast cancer survivors are also at a higher risk of developing post-treatment cardiac diseases [14, 15]. Inhibitors of the human epidermal growth factor-2 (HER-2) such as trastuzumab, are the standard of care for HER2-positive breast cancer [16]. The rate of cardiotoxicity in patients treated solely with trastuzumab is 3–7%, and it increases when trastuzumab is used in combination with anthracyclines, paclitaxel, and/or cyclophosphamide [17]. Trastuzumab cardiotoxicity is reversible, such that decreases in left ventricular ejection fraction (LVEF) can be used to direct treatment hiatus [16]. Cardiotoxicity arising from fluoropyrimidine treatment manifests as cardiomyopathy, pericarditis, and heart failure [18] but is also reversible upon cessation of therapy [19].

Radiation therapy is also commonly used in breast cancer therapy. Due to the anatomical proximity of the breast cancer lesions and the heart, the heart is at risk of irradiation. While historically the heart was considered to be resistant to doses of radiation below 30 Gy, several studies now indicate that radiation-induced heart disease can arise from much lower doses [20]. High radiation doses (> 30 Gy) can induce symptoms of heart disease within 1–2 years of exposure, while lower doses (< 20 Gy) may have a latency period of more than 10 years. Radiation-induced heart disease can manifest as pericarditis, myocardial fibrosis, and, potentially, coronary artery disease [21]. Fibrosis may be asymptomatic and detected only incidentally by echocardiography scanning more than 10 years after radiation therapy [22]. Multiple strategies for reducing the cardiac impact of external beam irradiation are implemented in planning and treatment programs, but the risk of cardiac toxicity is still considerable [23]. To maximize the success of these strategies requires more complete understanding of the pathophysiology of radiation-induced damage and therefore early detection of cardiac injury. This is challenging due to the long latency period between radiotherapy and the onset of symptoms of heart disease [20].

The financial, medical, and health costs of chronic heart disease in cancer survivors are significant [24–26]. The fact that cardiotoxicity can be reversible emphasizes the importance of early detection to allow appropriate treatment strategies to be implemented. However, clinical guidelines for monitoring cardiotoxicity during and after cancer therapies are currently lacking [27]. Cardiotoxicity is broadly defined in terms of measurable changes in cardiac function and assessed by measurement of LVEF, but this measure is operator-dependent [28], and a poor index of early myocardial damage [11, 28, 29], that does not adequately predict subsequent declines in function [11]. Moreover, a sizeable proportion of heart failure patients present with preserved LVEF [30]. Routine echocardiographic screening of survivors of childhood cancer increases life expectancy and quality-of-life years relative to no screening, but gains to date are modest [26]. Cardiac MRI using late gadolinium enhancement currently detects some cardiac injury, but the method has shown poor diagnostic and prognostic value [31]. Few cardiovascular biomarkers aside from circulating cardiac troponins and B-type natriuretic peptide can be detected non-invasively [31, 32], but the sensitivity and specificity of these biomarkers may be insufficient to enable their use as single predictive markers [33].

**Fig. 1:**
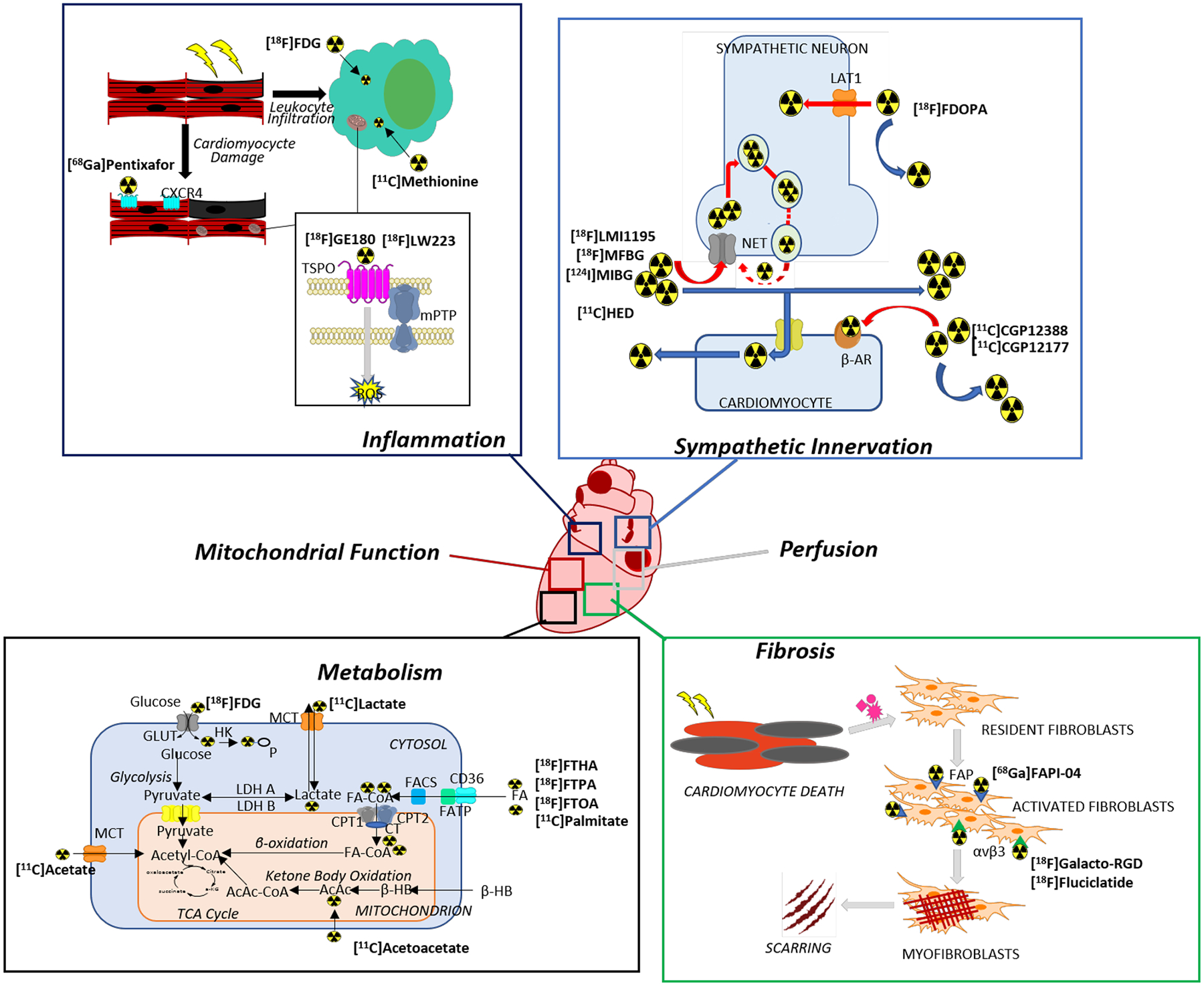
Overview of cardiac functions and pathophysiological pathways in cardiotoxicity for which emerging PET tracers are proposed to have diagnostic or prognostic function. Leading examples of tracers for each of pathway are discussed in the following sections and their molecular or biochemical targets are indicated in the panels. Abbreviations: CXCR4 = C-X-C chemokine receptor type 4; TSPO = 18-kDa translocator protein; mPTP = mitochondrial permeability transition pore; ROS = reactive oxygen species; LAT = L-amino acid transporter; NET = norepinephrine transporter; β-AR = β-adrenergic receptor; FAP = fibroblast activation protein; GLUT = glucose transporter; HK = hexokinase; MCT = monocarboxylate transporter; LDH = lactate dehydrogenase; FA = fatty acid; FATP = fatty acid transport protein; FACS = fatty acid-CoA synthetase; CPT = carnitine palmitoyltransferase; CT = carnitine acyltransferase; TCA = tricarboxylic acid; AcAc = acetoacetate; β-HB = β-hydroxybutyrate

Nuclear imaging with radiopharmaceuticals is emerging as a method of imaging cardiac function and pathology. It is now possible to image pathological processes, such as fibrosis, inflammation, metabolism, mitochondrial function, perfusion, and sympathetic innervation, using radiolabeled probes (Fig. 1). Assessment of myocardial perfusion using single photon computed tomography (SPECT) is now routine [34], but the proliferation of cameras for positron emission tomography (PET) as well as probes labeled with short-lived radioisotopes that can be incorporated into substrates or substrate analogues of numerous biological pathways make this technology a viable tool for cardio-oncology [35, 36]. In comparison to SPECT imaging, PET imaging benefits from higher count sensitivity, robust attenuation correction, and enhanced anatomical information via hybrid PET/CT and PET/MRI devices [35]. Given these characteristics, PET imaging can be exploited for imaging individual biochemical pathways implicated in numerous cardiac pathophysiologies. In this review, we discuss emerging PET probes for imaging incipient cardiac damage arising from cancer therapy.

## Myocardial Perfusion

Myocardial perfusion imaging remains the most common application of radionuclide imaging in nuclear cardiology and cardio-oncology. Perfusion imaging measures coronary microvascular function, which may be disrupted in patients with coronary artery disease [37]. Both [^82^Rb]Rb^+^ and [^13^ N]ammonia are routinely used in clinical practice for quantification of myocardial perfusion and myocardial perfusion reserve. A newer agent labeled with fluorine-18, [^18^F]flurpiridaz, recently completed a phase III trial in which coronary artery disease was detected with greater sensitivity than ^99m^Tc SPECT [38]. [^18^F]Flurpiridaz potentially exploits the superior physical properties of fluorine-18 compared to nitrogen-13 and rubidium-82. Recently, these tracers have found application in cardio-oncology to image compromised heart function as a result of cancer therapy. Myocardial perfusion reserve as assessed by [^82^Rb]Rb^+^ PET decreased following doxorubicin exposure in a cohort of lymphoma patients [37]. A similar finding was recently reported for breast cancer patients receiving radiotherapy using [^13^ N]ammonia PET [39]. The prognostic value of these changes in myocardial perfusion reserve is yet to be established.

## Mitochondrial Dysfunction

Doxorubicin accumulates in mitochondria over time [40]. Accumulation of doxorubicin may damage mitochondria through disruption of the electron-transport chain and the generation of free radicals [41, 42]. Given the enrichment of mitochondria in cardiomyocytes, selective damage to mitochondria may be a major mechanism of cardiotoxicity induced by doxorubicin. One method of assessing mitochondrial function is to measure the mitochondrial membrane potential. Mitochondrial membrane potential reflects aerobic energy production as well as other mitochondrial functions such as Ca^2+^ import or NADPH generation [43]. Membrane potential can be quantitated by measuring the distribution of radiolabeled probes. For example, lipophilic cationic agents used to image cardiac perfusion are also sensitive to changes in the mitochondrial membrane potential [44]. More than 90% of the cardiac accumulation of ^99m^Tc-MIBI is due to its trapping in mitochondria [45, 46]. On this basis, radiolabeled cations have been proposed to be tools for non-invasive assessment of mitochondrial dysfunction arising from cardiotoxicity [46]. Loss of ^99m^Tc-MIBI retention is evident in preclinical models of acute cardiotoxicity [44]. A recent study highlights the possibility of visualizing disrupted mitochondrial membrane potential by PET. In this study, cardiac retention of a radiolabeled phosphonium cation, [^18^F]MitoPhos, decreases in a dose-dependent manner in rat hearts following doxorubicin treatment [47]. The decrease in retention in the left ventricle was nearly twofold in comparison to controls and preceded detectable changes in cardiac troponins. A similar reduction in the cardiac retention of [^68^ Ga]Galmydar, a P-glycoprotein substrate that localizes in the mitochondria of cardiomyocytes, was also reported [48], reinforcing the promise of radiolabeled lipophilic cations for detection mitochondrial dysfunction. Future clinical evaluations are required to determine the sensitivity of this technique to anthracycline-induced cardiotoxicity.

## Innervation

Serial measurement of LVEF is the gold standard for assessing cardiac function, but a sizeable proportion of heart failure patients present with preserved LVEF [30]. Activation of the cardiac nervous system is partly responsible for preservation of LVEF [49] and is therefore a pathway of interest for nuclear imaging of cardiac function. Cardiac sympathetic activation is potentially beneficial in early stages of heart failure, but its longer-term activation is detrimental [50]. Consequently, sympathetic cardiac nerve dysfunction is characteristic in cardiomyopathy, heart disease [51], and heart failure [52]. To date, multiple molecular imaging probes for imaging presynaptic neurons are in clinical use or under clinical evaluation. Norepinephrine is the major neurotransmitter of the sympathetic nervous system. Metaiodobenzylguanidine (MIBG) is a norepinephrine analogue that targets the norepinephrine transporter (NET) and shares common pathways of uptake, storage, and release with norepinephrine in presynaptic sympathetic neurons of the myocardium [50]. On this basis, radioiodinated MIBG is used for both imaging and targeted radiotherapy applications in the clinical setting. [^123^I]MIBG scintigraphy is used for tumor imaging [53], neurodegenerative disease [54], and imaging cardiac events in heart failure [55]. Deficits in cardiac [^123^I]MIBG uptake are evident in preclinical models of anthracycline-induced cardiotoxicity [56, 57]. Uptake also decreases in a dose-dependent manner in patients undergoing doxorubicin chemotherapy [58, 59]. In these patients, decreases in uptake precede deterioration of LVEF, but predict subsequent decreases in heart function and progression to heart failure [59].

The clinical success of [^123^I]MIBG has yet to be translated to a corresponding PET probe, but positron-emitting analogues such as [^124^I]MIBG and [^18^F]MFBG are currently in clinical evaluation for imaging neuroblastoma [60–62]. These early studies confirm high myocardium-to-mediastinum ratios (MMR) in normal rodent and human hearts. A potential benefit of [^18^F]MFBG relative to [^123/124^I]MIBG is the decrease in radiation dose due to the shorter half-life of fluorine-18 (t _1/2_ = 110 min) and avoidance of radioactivity accumulation in the thyroid [60]. Moreover, the uptake of [^18^F]MFBG corresponds with NET expression [63]. These characteristics also translate favorably to application to myocardial PET imaging. [^18^F]LMI1195, a radiofluorinated NET substrate, also exploits the favorable physical properties of fluorine-18. In preclinical models, [^18^F]LMI1195 uptake and retention in the healthy myocardium is comparable to [^123^I]MIBG with improved contrast to normal tissue and high sensitivity to denervation [64, 65]. [^18^F]LMI1195 has completed phase 1 [66] and phase 2 [67] clinical trials and [^18^F]MFBG is currently in Phase 1/2 trials for imaging myocardial sympathetic innervation [68], leading to the possibility of image cardiac denervation in the clinic by PET in the near future.

An alternative to imaging NET expression and activity in heart failure is to image the utilization of radiolabeled norepinephrine analogues by PET. Hydroxyephedrine is one such synthetic analogue that accumulates in nerve terminals and is transported into neurons by NET. Within neurons, hydroxyephedrine is transported into storage vesicles. Preclinical studies confirm that meta-[^11^C]hydroxyephedrine ([^11^C]HED) is characterized by high selectivity, long neuronal retention times, likely due to dynamic recycling of [^11^C]HED by cardiac sympathetic neurons, and high correlation with tissue norepinephrine concentrations. The recycling of this probe by cardiac sympathetic neurons likely renders its retention in neurons sensitive to changes in nerve density, NET activity, vesicular storage, and possibly sympathetic nerve activity [69]. This is demonstrated by numerous clinical imaging studies, which confirm global and regional decreases in [^11^C]HED uptake in patients with dilated cardiomyopathy [70], acute myocardial infarction [71], and congestive heart failure [72]. Similar observations were noted in a cohort of patients with coronary artery disease but no history of myocardial infarction [73], demonstrating the broad applicability of [^11^C]HED PET to multiple cardiac pathologies. Regional variation in [^11^C]HED uptake in patients with ischemic cardiomyopathy predicted cause-specific mortality from sudden cardiac arrest [74], thereby validating PET imaging of cardiac sympathetic innervation as a predictive biomarker. These studies also indicate that the volume of [^11^C]HED deficit is independent of LVEF and infarct volume, a finding that reinforces earlier preclinical studies [75]. Notwithstanding these encouraging studies, the requirement for an on-site cyclotron to produce carbon-11 restricts [^11^C]HED to a research setting.

[^18^F]FDOPA broadly facilitates study of the striatal dopaminergic system, but its primary clinical application is for imaging brain [76] and neuroendocrine tumors [77]. In contrast to [^11^C]HED, [^18^F]FDOPA is suitable for a centralized production and distribution paradigm. The tracer is increasingly recognized to be capable of targeting pathways in multiple disorders [78], and recent feasibility studies demonstrate its utility in imaging cardiac sympathetic denervation. In one group of patients with clinically diagnosed heart failure, regional and global deficits in [^18^F]FDOPA uptake, defined by decreasing MMR, relative to healthy controls [79]. Additionally, [^18^F]FDOPA documents cardiac sympathetic dysfunction in patients with advanced idiopathic Parkinson’s disease [80].

Prolonged cardiac sympathetic activation downregulates postsynaptic β-adrenergic receptors [81]. This decrease in receptor density and occupancy has been measured in patients with nonischemic cardiomyopathy using the radiolabeled beta-blockers [^11^C]CGP12388 [82] and [^11^C]CGP12177 [83]. Assessment of β-adrenergic receptor density by this means highlights a correlation between density decrease and functional improvement after beta-blocker therapy [84], suggesting the possibility of using PET imaging to identify heart failure patients that would best benefit from beta-blockers.

## Inflammation

Any cardiac insult may induce inflammation, which is required to commence healing [85]. When the inflammatory response is dysregulated, resulting in either excessive or insufficient activity, then inflammation itself may contribute to tissue damage and a poorer outcome. This pathological response is thought to contribute to ventricular remodeling and adverse outcomes following myocardial infarction [86], and to accelerating damage in nonischemic cardiomyopathy as well as promoting progression to heart failure [87]. Direct infiltration of immune cells may also be a cause of cardiomyopathy [85]. Inflammation is also a hallmark of cardiotoxicity arising from anthracycline therapy [88]. The inflammatory response may be evident shortly after cardiac injury [85]. As inflammation is therefore both an index of myocardial damage and a potential opportunity for therapeutic intervention, it represents an attractive target for molecular imaging in nuclear cardiology and cardio-oncology.

There are no consensus imaging biomarkers for inflammation arising from cancer therapy. One strategy is to image immune cells that infiltrate the myocardium. Inflammatory leukocytes demonstrate elevated metabolic activity characterized by increased glucose utilization [89]. [^18^F]FDG is taken up avidly by metabolically-activate macrophages [90]. Consequently, recruitment of these cells as part of the inflammatory response leads to increased accumulation of [^18^F]FDG as visualized by PET. This was confirmed by preclinical studies in a coronary ligation-induced infarction model, for which [^18^F]FDG uptake peaked 5 days post-infarct and corresponded to inflammatory regions populated by CD11b + monocytes [91]. Uptake post-infarct is typically highest in the border zone of the infarcted myocardium [92] despite recruitment of inflammatory mediators to the remote myocardium [91]. These findings were reinforced in post-acute myocardial infarction patients, where post-infarct [^18^F]FDG uptake also correlated inversely with longer term functional outcome [93].

The use of [^18^F]FDG for imaging cardiac inflammation is complicated by high myocardial uptake under physiological conditions. Interpretation of the signal is further compromised by the presence of damaged cardiomyocytes whose metabolism may be reprogrammed. Pre-scan myocardial suppression protocols attempt to suppress the contribution of cardiomyocytes to [^18^F]FDG uptake, but these protocols are not always effective and may manipulate the metabolism of ischemic cardiomyocytes in ways that have yet to be determined [94]. These challenges have sparked the search for new targets that would allow higher contrast between the inflamed and healthy myocardium. Methionine is taken up by inflammatory leukocytes but is not a major metabolic substrate for the healthy myocardium [95]. On this basis, [^11^C]methionine PET has been investigated in preclinical models of myocarditis and myocardial infarction. Uptake of the probe in rat hearts colocalized with histologically-proven inflammatory regions, although the contrast to non-inflammatory lesions was lower than was achieved with [^18^F]FDG [96]. Baseline [^11^C]methionine uptake in mouse hearts was indistinguishable from background, but uptake significantly increased the infarct area and corresponded to infiltration of macrophages into the damaged myocardium [97]. In a small cohort of patients with acute myocardial infarction, [^11^C]methionine uptake increased in the infarcted region while [^18^F]FDG uptake decreased [98]. Further studies in larger patient groups may better define the prognostic value of [^11^C]methionine inflammation imaging in the context of decreased cardiac function.

A growing number of molecular targets for imaging have been identified within cardiomyocytes and infiltrating immune cells that contribute to the inflammatory response. Translocator Protein 18 kDa (TSPO) is expressed in the mitochondria of cardiomyocytes, where it is proposed to play a key role in regulating cardiac function [99]. Expression is sensitive to inflammation, and increases during myocarditis [100, 101] and cardiac hypertrophy induced by transverse aortic constriction (TAC) [102], where the increase is associated with oxidative stress, metabolic failure, and systolic dysfunction [102, 103]. TSPO may regulate the mitochondrial permeability transition pore (mPTP), which opens following mitochondrial swelling induced by infarction-reperfusion injury [104]. mPTP opening subsequently leads to necrosis and apoptosis. TSPO is also recognized as a marker of activated macrophages [105] that infiltrate the myocardium following pressure overload [84]. Preclinical rodent models confirm elevated uptake of the radiolabeled TSPO ligands [^18^F]GE180 [95, 106••] and [^18^F]LW223 [107] in the infarct region by 7 d post-infarction in concert with increases in TSPO expression. Early increases in TSPO ligand signal predict subsequent left ventricular remodeling [95] and progression to cardiomyopathy and heart failure [108]. Strikingly, the severity of inflammation at 7 d post-infarction coincides with increased [^18^F]GE180 uptake even at sites distal to the infarct and that do not exhibit infiltrating inflammatory cells [95, 109]. Moreover, [^18^F]GE180 signal in the remote myocardium is elevated at 8 weeks post-infarction, which is taken to be evidence of mitochondrial stress [106••]. TSPO expression is suppressed by doxorubicin [110], and treatment of isolated cardiomyocytes with TSPO ligands reduces doxorubicin-induced deficits in cardiac contractility [111]. These findings reinforce an independent role for TSPO in the progression of cardiomyopathy [112] and as an imaging target in cardio-oncology.

CXCR4 and its ligand CXCL12 mediate immune cell recruitment and are involved in multiple inflammatory conditions. In progressive atherosclerosis and acute myocardial infarction, recruitment of leukocytes to the injured region by the CXCR4/CXCL12 pair is thought to be a component of the healing response [113]. This is supported by evidence from genetic and chemical preclinical models that knockout or continuous blockade of CXCR4 exacerbates cardiac dysfunction and promotes cardiomyopathy [114, 115]. Notwithstanding these data, a single dose of antagonist at an early time point improves healing and recovery of function [116]. In this context, imaging CXCR4 expression is a promising strategy for early detection of inflammation and immune response to cardiac insult. It was recently shown that [^68^ Ga]Ga-pentixafor, a high affinity antagonist of the CXCR4 receptor, accumulates in the left ventricle of mice in the 7 days following transverse aortic constriction [117]. Additionally, CXCR4-specific [^68^ Ga]Ga-pentixafor uptake increased in the infarct region of the myocardium in an experimental model of acute myocardial infarction. In both cases, uptake correlated with infiltration of CD45 + leukocytes and CD68 + macrophages [118]. Tracer uptake peaked at day 3 post-infarct and subsequently declined. Similar findings were reported following administration of a ^68^ Ga-labeled bifunctionalized recombinant murine CXCL12 probe [119]. In human acute myocardial infarction patients, changes in [^68^ Ga]Ga-pentixafor uptake in infarct regions were variable [118], but negatively correlated to scar volume at follow-up [120]. These findings suggest that CXCR4 imaging may be valuable immediately after cardiac insult to assess the size of the injured area and predict healing.

## Fibrosis

Myocardial fibrosis is a crucial component of cardiac dysfunction arising from cancer therapy [2, 121, 122]. Fibrosis results from the invasion of activated fibroblasts into the myocardium, with subsequent remodeling of the extracellular matrix (ECM) leading to formation of regions of reduced elasticity [123]. If the healing process is incompletely resolved, the heart undergoes adverse left ventricle remodeling [124]. Subsequently, the heart might experience dysfunction and be vulnerable to arrhythmias or ischemia [125]. These same processes also occur following myocardial infarction [124, 126]. Biopsy is currently the gold standard for diagnosis of myocardial fibrosis [125]. Although multiple circulating biomarkers have been proposed as non-invasive means of diagnosing myocardial fibrosis, to date, only the carboxy-terminal propeptide of procollagen type I and amino-terminal propeptide of procollagen type III correlate to the collagen volume fraction [125]. Moreover, the tissue origin of these circulating enzymes cannot be determined, limiting their diagnostic accuracy.

The central role of cardiac fibroblasts in fibrosis and cardiomyopathy is well documented: a decrease in cardiomyocytes induces activation of cardiac fibroblasts and formation of a collagen scar [127]. Scarring may decrease heart muscle function and is often only visible by echocardiography after function is already compromised. Detection of this pathophysiological process at an early stage requires identification of a specific biomarker. Fibroblast activation protein alpha (FAP) may serve this role [128]. FAP is a transmembrane serine protease involved in ECM remodeling and cell migration that is expressed almost exclusively in activated fibroblasts [129]. Myocardial FAP expression increases during cardiac remodeling [129–132], but activated fibroblasts are not evident in mature scars [133]. Preclinical studies confirm the uptake of radiolabeled FAP inhibitors in the infarct region and border region of rodent hearts [126, 134]. In these models, peak uptake is reached 7 days post-infarction, with signal evident until 21 days [126]. Signal was confirmed histologically to correspond to FAP expression.

The majority of clinical FAP inhibitor PET scans have thus far been performed in cancer patients, but anecdotal observations of increased tracer uptake in patients with concomitant coronary artery disease attest to the potential for this technique in detecting cardiac dysfunction. [^68^ Ga]FAPI-04 is a ^68^ Ga-labeled small molecular FAP inhibitor that has been evaluated in 28 different human cancers [135]. Intense, focal uptake of [^68^ Ga]FAPI-04 in the infarct area post-myocardial infarction is anecdotally noted in a number of human patients [133, 136, 137]. In these images, [^68^ Ga]FAPI-04 accumulates to a minimal extent in the remote myocardium, with uptake comparable to control subjects with no cardiac damage [133]. [^68^ Ga]FAPI-04 PET overestimates the infarct area, likely due to activation of fibroblasts in the infarct border zone, but no signal is evident in mature scars [133]. The specificity of [^68^ Ga]FAPI-04 for activated fibroblasts may allow this probe to serve as a diagnostic biomarker. This is supported by a recent clinical report of a patient retrospectively being diagnosed with a myocardial infarction by echocardiography after intense cardiac uptake of [^68^ Ga]FAPI-04 [136].

A clinical report of intense [^68^ Ga]FAPI-04 accumulation in the left ventricular myocardium in a cancer patient with a history of coronary artery disease but no signs of chronic nor acute coronary syndromes identifies a possible role for this probe in the early detection of myocardial damage during cancer therapy [138]. A retrospective analysis of patients who received [^68^ Ga]FAPI-04 as part of cancer staging identified a correlation between myocardial uptake of this probe and cardiac injury [139]. These findings were confirmed in a larger retrospective study of cancer patients imaged with [^68^ Ga]FAPI-04, which demonstrated a correlation between signal intensity in the myocardium and cardiovascular risk factors, including a history platinum-based chemotherapeutics or chest irradiation [140••]. These studies highlight the potential utility of [^68^ Ga]FAPI-04 to noninvasive detection of cardiotoxicity. Larger, prospective studies will help to define its specific value in monitoring cardiac health during cancer therapy.

Activated cardiac fibroblasts also express α_v_β_3_ integrin during pathophysiologies that lead to extracellular matrix remodeling [141]. This expression complements α_v_β_3_ upregulation in vascular endothelial cells within the myocardium during states of angiogenesis post-infarction [142, 143]. Together, these observations support a central role for α_v_β_3_ integrin in angiogenesis and coordinating tissue repair after myocardial infarction [143]. On this basis, PET imaging using probes that target α_v_β_3_ integrin represents a possible method of visualizing cardiac tissue repair. In a preclinical model of coronary artery occlusion followed by reperfusion, focal uptake of the high affinity antagonist [^18^F]F-Galacto-arginine-glycine-aspartate (RGD) peaked between 1 and 3 weeks in the infarct area before decreasing [142]. These findings were supported by a recent study in a cohort of patients with acute myocardial infarction [143]. Focal myocardial uptake of [^18^F]fluciclatide, a high affinity RGD-derived ligand, in these patients colocalized to the infarct region. Moreover, α_v_β_3_ integrin expression, as determined by [^18^F]fluciclatide PET, increases in aortic atherosclerotic plaques, and this is particularly evident in patients with recent myocardial infarction [144]. These preliminary studies identify α_v_β_3_ integrin as a potential imaging biomarker of inflammation and cardiac remodeling, though studies in larger cohorts are necessary to determine the sensitivity and prognostic value of this candidate biomarker.

## Metabolism

The majority of energy metabolism in the healthy heart is oxidative phosphorylation, which supplies more than 95% of ATP generated in the heart [145]. The major driving force of this ATP production, supplying approximately 70%, is β-oxidation of fatty acids. The remaining supply of ATP is produced from oxidation of glucose, lactate, or ketone bodies. Experimental models indicate that fatty acid utilization substantially decreases in hypertrophy, cardiomyopathy and heart failure. This may result in a compensatory increase in glucose oxidation, but results published to date are inconsistent in this regard. A confounding factor may be the increase of anaplerosis, which diverts pyruvate away from mitochondrial oxidation and potentially decouples glycolysis and glucose oxidation. Preclinical and preliminary clinical studies using radiolabeled myocardial metabolic substrates or substrate analogues support the application of metabolic PET imaging to detection of myocardial ischemia, assessment of viability, and early detection of cardiomyopathy [146].

Radiolabeled fatty acids and fatty acid analogues have long been investigated for noninvasive measurement of myocardial metabolism in the context of ischemia and cardiomyopathy [147]. [^11^C]Palmitate is rapidly taken up homogenously by healthy human and animal hearts [148], and its washout is proposed to reflect both fatty acid β-oxidation (rapid) and incorporation into the cardiomyocyte triglyceride pool (slow) [146]. Under ischemic conditions or during ischemic dilated cardiomyopathy, [^11^C]palmitate uptake decreases [149, 150]. This is attributed to diminished capacity of the myocardium to extract, retain, and metabolize long-chain fatty acids [151] and enables delineation of the infarct area based on regional defects of [^11^C]palmitate uptake [149]. By contrast, the myocardial distribution of [^11^C]palmitate is more heterogenous in patients with nonischemic dilated cardiomyopathy [152]. The acquisition of dynamic PET images following administration of [^11^C]palmitate enables myocardial fatty acid utilization and myocardial fatty acid oxidation to be qualitatively estimated after application of a suitable kinetic model. Nevertheless, the distribution of radiolabeled fatty acids into multiple lipid pools with different kinetics complicates the measurements of uptake and myocardial metabolism kinetics [147, 148]. Both a four-compartment model [153–155] and a three-tissue compartment model [156, 157] have been proposed for analysis of the kinetic data. To date, the majority of these kinetic analyses have been carried out in animal models, highlighting the knowledge gap that must be bridged before these probes become clinically routine.

[^18^F]Fluoro-6-thia-heptadecanoic acid, [^18^F]FTHA, utilizes a sulfur atom in the fatty acid backbone to inhibit β-oxidation [158]. In contrast to [^11^C]palmitate, this probe is not fully metabolized and therefore quantitation of uptake is possible after correcting the arterial input function for plasma metabolites [147]. By comparison to [^11^C]palmitate and 17-[^18^F]fluoroheptadecanoic acid [159], myocardial uptake of [^18^F]FTHA is similar, but its clearance is significantly slower [158]. The rate of [^18^F]FTHA uptake increases in patients with congestive heart failure [160]. The second- and third-generation analogues 16-[^18^F]fluoro-4-thia-palmitate and 18-[^18^F]fluoro-4-thia-oleic acid, respectively, have increased specificity to myocardial fatty acid oxidation and improved myocardial retention [161, 162]. Recent in vitro studies confirm that these next-generation ^18^F-fluorinated thia-oleic acid derivatives are also taken up by fatty acid transporters and are retained in cardiomyocytes after becoming metabolically blocked in the β-oxidation pathway [163].

More recent studies also identify a possible role for metabolic PET imaging in assessing cardiotoxicity arising from cancer treatment [164–167]. As energy production is essential to cardiac function, the metabolic pathways that drive this process represent promising candidates for early identification of cardiotoxicity. [^18^F]FDG is a radiolabeled glucose analogue that is trapped in cells following its phosphorylation by hexokinase. Consequently, it is used to assess myocardial glycolytic flux. A reduction in β-oxidation may induce a compensatory increase in glycolysis that could be visualized non-invasively by [^18^F]FDG PET. Increased glucose utilization, as indicated by increased [^18^F]FDG uptake, is evident in rat hearts following treatment with sunitinib [164]. Moreover, cardiac uptake of [^18^F]FDG increases in lung cancer [166] and esophageal cancer [167] patients treated with radiotherapy and in lymphoma patients treated with anthracyclines [168•]. However, while [^18^F]FDG signal increase correlated with LVEF decrease in these patients, the studies were largely conducted retrospectively. Prospective and long-term studies are needed to confirm an association between early increase in myocardial [^18^F]FDG signal and later loss of function [169].

^11^C-Labeled metabolites are potentially valuable alternatives to [^18^F]FDG because they are fully metabolized by the heart and can therefore allow the rates of metabolic processes to be qualitatively estimated. The short half-life of carbon-11 (t_1/2_ = 20.8 min) contributes to reducing the radiation dose, thereby facilitating serial scanning of patients with suspected cardiotoxicity. Typically, the rate of utilization of these probes is decreased as a consequence of metabolic impairment in the failing heart. [^11^C]Acetate is a tool for measuring myocardial oxygen consumption because it is readily oxidized to [^11^C]CO_2_ [170]. Regional decreases in oxidative metabolism are evident by [^11^C]acetate PET in patients with hypertrophic cardiomyopathy [171, 172]. Overlapping pathologies between this condition and cardiotoxicities suggest a role for [^11^C]acetate in the cardio-oncology setting. To this end, a decreased rate of myocardial [^11^C]acetate utilization is evident in a preclinical model of sunitinib cardiotoxicity [164]. Similarly, [^11^C]acetoacetate, a ketone body, is not utilized to the same extent in the myocardium of rats with doxorubicin-induced cardiotoxicity as in healthy controls [165]. Finally, as the heart uses lactate as a source of acetyl-CoA to supply the tricarboxylic acid cycle [158], [^11^C]lactate is proposed to be a potential probe for assessing myocardial metabolism by PET [173]. Feasibility studies in animal models confirm that [^11^C]lactate PET correlates with lactate oxidation and support the addition of this probe to the toolbox of myocardial metabolic imaging agents. Despite these promising preclinical studies, the clinical application of these ^11^C-labeled probes is currently limited by the need to perform complex metabolite analysis to derive a suitable input function for kinetic modeling [148]. Nevertheless, the availability of these ^11^C-labeled probes by reliable chemistry combined with the ever-improving capabilities of clinical PET scanners supports further evaluation of metabolic imaging in assessing cardiac health and function during cancer therapy.

## Conclusions and Further Perspectives

Cardiotoxicity encompasses a broad range of pathophysiological process that may ultimately lead to heart failure. Although the precise mechanisms by which cancer treatment causes cardiac damage are still being elucidated, it is likely that one or more of restricted flow, inflammation, cardiac remodeling, sympathetic denervation, and metabolic dysfunction are involved. Appropriate modification of treatment to protect the heart could dramatically increase quality of life, reduce medical expenses, and even be life-saving. Notwithstanding the major clinical challenge that cardiotoxicity poses to cancer patients, there are no reliable methods of detecting incipient cardiac injury before the damage is irreversible. Therefore, there is both a great need and an unprecedented opportunity for molecular imaging probes to identify the pathophysiological processes that underpin cardiotoxicity even when patients present with preserved LVEF or normal echocardiographic scans. As many of these processes overlap with those that arise as a result of other chronic or acute myocardial insults, the evaluation of new PET tracers in patients with heart failure promises to be of great value to cardio-oncology. In this review, we highlight the preclinical and clinical evidence for markers of perfusion, mitochondrial function, inflammation, fibrosis, and cardiomyocyte metabolism as diagnostic and predictive biomarkers of cardiac damage.

Among the tracers to have recently undergone clinical evaluation, radiolabeled inhibitors of fibroblast activation protein are particularly promising early markers of cardiac injury. Multiple pathways contribute to cardiomyocyte death, but cardiomyopathy is ultimately the result of functional decline of the heart due to stiffening and scarring of the muscle. Fibrosis is a key element of cardiomyopathy. Radiolabeled FAP inhibitors are synthesized in high radiochemical yields and purities and accumulate in the activated fibroblasts responsible for initiating the pathological healing response. In preliminary clinical studies, these compounds minimally accumulate in healthy tissue and rapidly clear from blood. Myocardial uptake is not restricted to the specific site of injury, e.g., the infarct region, but rather reflects the regional activation of fibroblasts that contribute to scarring. Furthermore, high signal at early time points predicts later stage cardiac remodeling. On the basis of these preliminary studies, FAP appears to be both a diagnostic and predictive biomarker. FAP imaging, therefore, may be transformational in nuclear cardiology and cardio-oncology.

Finally, technological innovation in imaging physics and camera design may promote new probes to the fore-front of PET imaging in cardiology and cardio-oncology. As the sophistication of PET cameras increases the metabolic information that can be extracted from imaging studies with ^11^C-labeled polar metabolites, such as [^11^C]acetate or [^11^C]lactate, may become increasingly detailed. In principle, metabolic PET imaging is not only a real-time snapshot of cardiac metabolism but also a tool for quantifying the rates of specific metabolic pathways relevant to cardiac health. The challenge of identifying a suitable kinetic model has, to date, rendered these ^11^C-labeled probes qualitative rather than measures of cardiac metabolism. Whole body scanners offer the possibility of determining the input function from a single scan rather than complex metabolite analysis, thereby addressing a major limitation of these probes. The requirement for carbon-11 likely restricts the use of probes such as [^11^C]lactate or [^11^C]acetoacetate to the research setting, but the biochemical information extracted from these ^11^C-labeled probes may complement information obtained from other imaging modalities such as SPECT and hyperpolarized magnetic resonance imaging [174] and suggest new probes labeled with fluorine-18 for assessing myocardial metabolism in large clinical populations. With these tools in hand, a renaissance in metabolic imaging as a way of non-invasively assessing cardiac health in cardio-oncology is a distinct possibility.
